# The causal relationship between O2:K7:H6 extra-intestinal pathogenic *Escherichia coli* (ExPEC) and native valve endocarditis: a case report

**DOI:** 10.1186/s12879-021-06066-y

**Published:** 2021-04-20

**Authors:** Marie-Françoise Leonard, Hector Rodriguez-Villalobos, Nadia Boisen, Flemming Scheutz, Pierre-François Laterre, Philippe Hantson

**Affiliations:** 1 Laboratoire Synlab, 4020 Liège, Belgium; 2 Department of Microbiology, Cliniques universitaires St-Luc, Université catholique de Louvain, Brussels, Belgium; 3 Department of Bacteria, Parasites and Fungi, Statens Serum Institut, The International Centre for Reference and Research on Escherichia and Klebsiella, Copenhagen, Denmark; 4 Department of Intensive Care, Cliniques universitaires St-Luc, Université catholique de Louvain, Brussels, Belgium

**Keywords:** Native mitral valve, Endocarditis, *Escherichia coli*, Urinary tract infection - virulence factors, Case report

## Abstract

**Background:**

Native valves infective endocarditis due to *Escherichia coli* is still a rare disease and a particular virulence of some *E.coli* isolate may be suspected.

**Case presentation:**

A 79-year-old woman presented during the post-operative period of an orthopedic surgery a urinary tract infection following obstructive ureteral lithiasis. *E. coli* was isolated from a pure culture of urine and blood sampled simultaneously. After evidence of sustained *E.coli* septicemia, further investigations revealed acute cholecystitis with the same micro-organism in biliary drainage and a native valve mitral endocarditis. *E.coli* was identified as O2:K7:H6, phylogenetic group B2, ST141, and presented several putative and proven virulence genes. The present isolate can be classified as both extra-intestinal pathogenic *E.coli* (ExPEC_JJ_) and uropathogenic *E. coli* (UPEC_HM_).

**Conclusions:**

The relationship between the virulent factors present in ExPEC strains and some serotypes of *E. coli* that could facilitate the adherence to cardiac valves warrants further investigation.

## Background

Native valves infective endocarditis due to *Escherichia coli* is still a rare disease, but can be observed with an increasing frequency among older women who usually present additional risk factors [1]. It usually develops as a complication of urinary tract infection, and in this case, caused by an *E. coli* strain harboring an array of virulence factors.

## Case presentation

A 79-year-old woman, with a medical past history of obesity (BMI 30.8 kg/m^2^) and arterial hypertension, was admitted to the orthopedic ward followed by the operating theater, for the reduction of a luxation of her right hip total prosthesis. In the immediate postoperative period, she presented fever (39 °C), dyspnea with low oxygen saturation, and paroxysmal atrial flutter that resolved after bisoprolol administration. The patient was confuse and developed a progressive alteration of consciousness that required transfer to the Intensive Care Unit (ICU). On admission in the ICU (3 days after orthopedic surgery), the patient had a Glasgow Coma Scale (GCS) score of 9/15 (E2V2M5). Physical examination revealed temperature 38.7 °C, regular tachycardia (120/min) with arterial blood pressure 136/60 mmHg. There was no evidence of lateralized neurological deficit. Routine laboratory investigations showed: CRP 330 mg/L, white blood cell count 12,800/mm^3^, creatinine 95 μmol/L. Urine analysis confirmed leukocyturia (3658/μL), and *Escherichia coli* was isolated from a pure culture of urine (> 100.000 CFU/mL) and blood sampled simultaneously. Abdomen echography found a moderate left hydronephrosis (pyelic cavity 18 mm) in relation with a ureteral lithiasis. A percutaneous left nephrostomy was successfully performed and antimicrobial therapy was initiated with intravenous cefuroxime (4500 mg/day). The brain computed tomography (CT) with angiography failed to reveal any lesion. The electroencephalogram was characterized by diffuse slowing, without evidence of epileptic activity. The patient developed neck stiffness and remained with altered consciousness. No septic embolic lesions were noted on the skin. The lumbar puncture was technically not feasible due to lumbar arthrosis. The brain magnetic resonance imaging (MRI) did not reveal brain abscesses, but well multiple ischemic embolic lesions. The analysis of the different MRI sequences concluded that these lesions were not recent. Despite appropriate antimicrobial therapy and drainage, *E. coli* septicemia persisted for more than four consecutive days. In front of persistent bacteremia, there was a suspicion of either an intra-abdominal infectious source (urinary or biliary) or of an infectious endocarditis. The abdomen CT (on the fourth day in the ICU) suspected acute cholecystitis and a percutaneous drainage was proposed; the bile returned positive for *E. coli*. There was no sign of persisting hydronephrosis. Additionally, a transoesophageal echocardiography (TEE) was performed 3 days after sepsis onset and demonstrated two vegetations (8 mm for the greatest) on the anterior leaflet of the mitral valve and its chordae tendineae, with a mild mitral insufficiency, and the diagnosis of infective endocarditis was determined. Coronary angiography did not reveal significant lesions. Intravenous ciprofloxacin (1200 mg/day) was added to the treatment and the patient underwent on day 10 a mitral valve repair with pericardial patch on the anterior leaflet. The total duration of the combined antimicrobial therapy (cefuroxime and ciprofloxacine) was six weeks. The mitral valve endocarditis was confirmed by the inspection during the procedure, with also mitral valve calcifications. The culture of the native valve remained sterile. Histopathology confirmed the presence of a neutrophilic infiltrate and fibrinoid necrosis. Notably, there was a slow neurological improvement during the postoperative period. A second brain CT with angiography was performed 14 days after the first exam; a recent ischemic lesion was suspected in the right centrum semi-ovale. The patient was discharged from the ICU on day 28. Investigation by positron emission tomoscintigraphy (PET-FDG18) (performed on postoperative day 29) failed to demonstrate other peripheral embolization, and in particular no sign of infection on the site of the hip prosthesis. Two months later, the patient presented a new episode of *E. coli* septicemia due to the failure of the external drainage of the gallbladder. A new TTE confirmed the integrity of mitral valvuloplasty. At the six month follow-up, the patient was still dependent for daily life activities.

### Sero- and virulence typing of *E. coli* isolate C615–19

The *E. coli* isolate (C615–19), isolated from the patient, was whole genome sequenced (WGS) and phenotypically serotyped by The International *Escherichia* Centre (IEC) at Statens Serum Institut (Copenhagen, Denmark). Sequence type (ST), in silico serotype and virulence genes were determined from the de novo assembled genomes using the the *E. coli* plug-in in BioNumerics version 7.6.35510 (Applied Maths, Ghent, Belgium) and web tools available at https://cge.cbs.dtu.dk/services/ and an extended beta version of VirulenceFinder including 38 ExPEC related genes [2]. The O:K:H serotype was determined as being O2:K7:H6. Also WGS data analysis showed that the isolate belonged to phylogenetic group B2 and was ST141 and harboured 32 putative and proven virulence genes (Table 1). The antimicrobial resistance profile showed that C615–19 was resistant to ampicillin (MIC > 8 mg/L), piperacillin (MIC> 16 mg/L) and cotrimoxazole (MIC> 4/76 mg/L) as confirmed by the presence of the genes *bla*TEM-1, *sul2* and *dfrA5* (coding for ß-lactam, sulphonamide and trimethoprim resistance respectively). Furthermore, the isolate showed the presence of genes *strAa* and *strB* encoding kanamycin, neomycin, and streptomycin.

**Table 1:** Virulence genes found in C615–19

***Gene***	Description	Gene	Description	Gene	Description
*astA*	EAST-1 heat-stable toxin	*irp2*	Non-ribosomal peptide synthetase	*papA_F12*	Major pilin subunit
*chuA*	Outer membrane hemin receptor	*iss*	Increased serum survival	*papC*	Outer membrane usher P fimbriae
*cia*	Colicin ia	*iucC*	Aerobactin synthetase	*papGIII*	P adhesin allele III
*clbB*	Hybrid nonribosomal peptide	*iutA*	Ferric aerobactin receptor	*sfaD*	S fimbrial/F1C minor subunit
*cnf1*	Cytotoxic necrotizing factor	*kpsE*	Capsule polysaccharide export protein	*sfaE*	S fimbrial/F1C minor subunit
*focC*	S fimbrial/F1C minor subunit	*kpsM*	Polysialic acid transport protein	*sfaS*	S fimbriae minor subunit
*focI*	S fimbrial/F1C minor subunit	*mchB*	Microcin H47 part of colicin H	*traT*	Outer membrane protein
*fyuA*	Siderophore receptor	*mchC*	MchC protein	*usp*	Uropathogenic specific protein
*hlyA*	Hemolysin A	*mchF*	ABC transporter protein MchF	*vat*	Vacuolating autotransporter toxin
*hra*	Heat-resistant agglutinin	*mcmA*	Microcin M part of colicin H	*yfcV*	Fimbrial protein
*iroN*	Enterobactin siderophore receptor protein	*ompT*	Outer membrane protease		

## Discussion and conclusions

*E. coli* endocarditis remains a rare entity and represents approximately 0.51% of cases of infective endocarditis. It seems to be increasingly reported in older women [1, 3]. In most of the reported cases, endocarditis originated from a urinary tract infection and in a recent retrospective analysis, of the published cases, 52% of patients had a preceding urinary tract infection [1]. Additionally, it has been shown that some co-morbidities, such as diabetes, malignancy, excessive alcohol consumption, hemodialysis could predispose to *E. coli* infective endocarditis [1]. Several studies have reported, that among the four heart valves, the native mitral valve seems to be the most frequently affected endocarditis caused by *E. coli* [1, 4–10].. Here, we report a rare case of *E. coli* O2:K7:H6, B2, ST141 associated with urinary tract infection (UTI) and endocarditis. O2:H6, B2, ST141 Shiga toxin-producing *E. coli* (STEC) encoding Stx2b has been described as being closely related to adherent-invasive *E. coli* (AIEC) [11]. The heteropathogenic potential of these strains has been associated with their identification as diarrheagenic [11, 12] and their ability to cause urinary tract infection (UTI) in an animal model [11]. The serotype O2:H6 has been listed as isolated from UTI, bacteremia, ulcerative colitis, (bloody) diarrhea and meningitis in the IEC database, however, as of this case presentation, this is the only isolate with this particular serotype. Strains that have been fully O:K:H serotyped by the IEC are either O2:K1:H6 or O2:K7:H1 or O2:K7:H5. Our patient presented infective endocarditis of her native mitral valve, septic encephalopathy (with clinical signs of meningitis) and acute cholecystitis due to *E. coli* bloodstream infection secondary to hydronephrosis and urinary tract infection. How E.coli affects native valves is not completely known [1]. It is possible that specific virulence factors from extra-intestinal pathogenic *E.coli* (ExPEC) from the urinary tract may be strongly associated with the development of *E. coli* infective endocarditis. Studies have found that some *E. coli* strains with a high prevalence of phylogenetic type B2 could increase the probability for the pathogens to invade cardiac endothelia [13–15]. ExPEC are part of the gut microbiota of the healthy population but they are able also to produce disease after colonization of other non-intestinal niches. This pathogenesis has been related to their important genetic contents of virulence factors. Molecular epidemiological analyses have shown that ExPEC are distinct from commensal and diarrheagenic *E. coli* (DEC) in terms of pathogenic potential, ecology, evolution, reservoirs, transmission, pathways, host–pathogen interactions, and virulence mechanisms [16]. According to epidemiological and infection model data definitions the present isolate can be classified as both ExPEC_JJ_ [17] and uropathogenic *E. coli* (UPEC_HM_) [18]. Adhesins can play a key role in pathogenic process. As suggested by Nogueira et al. some of these factors could enable *E. coli* to adhere to cardiac valves [4]. The present isolate was positive for at least two adhesins, P fimbriae (*papA*_F12 and *papC*) and S fimbrial/F1C fimbriae (*focC, focI, sfaD, sfaE* and *sfaS*) that are frequent in uropathogenic human isolates and extraintestinal avian pathogenic *E. coli* (APEC) isolates. The WGS analysis showed that C615–19 harbored an array of ExPEC virulence (Table 1) factors such as the *iss* and *ompT* genes. Interestingly, both genes play a role in the ability of the bacterium to resist the innate host defenses. OmpT is active in the degradation of cationic peptides (defensins) excreted by epithelial cells from the urinary tract [19] and iss has been associated with increased serum survival [20] and found in *E. coli* isolated from female patients with bacteremia of urinary tract origin [21]. Little is known about the virulence genotypes of *E. coli* isolated from native valve endocarditis. However, one study [3] screened five *E. coli* strains for the presence of eight virulence genes, *papG, sfaS, ibeA, iucC, hly, cnf1, fyuA iroN*; they found that the strains harbored between three and five of the genes. Notably, C615–19 harbored all eight genes, except *ibeA* suggesting that it is a highly virulent strain which is also supported by our WGS analysis. Even though this *E. coli* strain is of a unique serotype, it shares virulence traits with ExPEC causing bacteremia and belongs to the most prevalent phylogenetic group, B2. These strains harbor multiple virulence factors that enable them to adhere, invade, escape host defenses and acquire essential nutrients, such as iron. C615–19 harbors all these factors. In conclusion, *E. coli* endocarditis is reported with increasing frequency, in particular among elderly patients, with high morbidity and mortality. The relationship between the virulent factors present in ExPEC strains and some serotypes of *E. coli* that could facilitate the adherence to cardiac valves warrants further attention to the typing of *E. coli*. It is possible, that *E. coli* O2:K7:H6 harboring this particular set of virulence genes may be associated with increased morbidity due to native valve endocarditis.

## Data Availability

All data and materials are available in the manuscript.

## Abbreviations

AIEC: Adherent-invasive *Escherichia coli*
APEC: Avian pathogenic *Escherichia coli*
BMI: Body mass index
CT: Computed tomography
GCS: Glasgow Coma Scale
ICU: Intensive care unit
ExPEC: Extra-intestinal pathogenic *Escherichia coli*
MRI: Magnetic resonance imaging
PET-FDG18: Positron emission tomoscintigraphy-^18^fluorodeoxyglucose
ST: Sequence type
STEC: Shiga toxin-producing *Escherichia coli*
UPEC_HM_: Uropathogenic *Escherichia coli*
UTI: Urinary tract infection
WGS: Whole gene sequencing

## References

[1] Akuzawa N, Kurabayashi M (2018). Native valve endocarditis due to *Escherichia coli* infection: a case report and review of the literature. BMC Cardiovasc Disord.

[2] Malberg Tetzschner AM, Johnson JR, Johnston BD, Lund O, Scheutz F. In Silico Genotyping of *Escherichia coli* Isolates for Extraintestinal Virulence Genes by Use of Whole-Genome Sequencing Data. J Clin Microbiol. 2020;22(58) e01269–20. 10.1128/JCM.01269-20 PMC7512150 32669379

[3] Micol R, Lortholary O, Jaureguy F, Bonacorsi S, Bingen E, Lefort A, Mémain N, Bouchaud O, Larroche C (2006). *Escherichia coli* native valve endocarditis. Clin Microbiol Infect.

[4] Nogueira AR, Brazão S, Ferreira D, Aragão A, Veríssimo MT, Carvalho A (2019). *Escherichia coli* endocarditis of a native mitral valve. IDCases.

[5] Menon T, Balakrishnan N, Somasundaram S, Dhandapani P (2017). Native valve endocarditis caused by *Escherichia coli*. J Clin Diagn Res.

[6] Chen CA, Lin ZZ, Yu WL, Wu WS (2015). *Escherichia coli* endocarditis of native aortic valve and mitral valve. J Formos Med Assoc.

[7] Rangarajan D, Ramakrishnan S, Patro KC, Devaraj S, Krishnamurthy V, Kothari Y, Satyaki N (2013). Native valve *Escherichia coli* endocarditis following urosepsis. Indian J Nephrol.

[8] Lauridsen TK, Arpi M, Fritz-Hansen T, Frimodt-Moller N, Bruun NE (2011). Infectious endocarditis caused by *Escherichia coli*. Scand J Infect Dis.

[9] Loubet P, Lescure FX, Lepage L, Kirsch M, Armand-Lefevre L, Bouadma L, Lariven S, Duval X, Yazdanpanah Y, Joly V (2015). Endocarditis due to gram-negative bacilli at a French teaching hospital over a 6-year period: clinical characteristics and outcome. Infect Dis (Lond).

[10] Kim CJ, Yi JE, Kim Y, Choi HJ (2018). Emphysematous endocarditis caused by AmpC beta-lactamase-producing *Escherichia coli*. Medicine (Baltimore).

[11] Bielaszewská M, Schiller R, Lammers L (2014). Heteropathogenic virulence and phylogeny reveal phased pathogenic metamorphosis in *Escherichia coli* O2:H6. EMBO Mol Med.

[12] Piérard D, Van Etterijck R, Breynaert J, Moriau L, Lauwers S (1990). Results of screening for verocytotoxin-producing *Escherichia coli* in faeces in Belgium. Eur J Clin Microbiol Infect Dis.

[13] Micenková L, Bosák J, Vrba M, Ševčíková A, Šmajs D (2016). Human extraintestinal pathogenic *Escherichia coli* strains differ in prevalence of virulence factors, phylogroups, and bacteriocin determinants. BMC Microbiol.

[14] Russo TA, Johnson JR (2000). Proposal for a new inclusive designation for extraintestinal pathogenic isolates of *Escherichia coli*: ExPEC. J Infect Dis.

[15] Micenková L, Beňová A, Frankovičová L, Bosák J, Vrba M, Ševčíková A, Kmeťová M, Šmajs D (2017). Human *Escherichia coli* isolates from Hemocultures: septicemia linked to urogenital tract infections is caused by isolates harboring more virulence genes than Bacteraemia linked to other conditions. Int J Med Microbiol.

[16] Johnson JR, Russo TA (2005). Molecular epidemiology of extraintestinal pathogenic (uropathogenic) *Escherichia coli*. Int J Med Microbiol.

[17] Johnson JR, Murray AC, Gajewski A, Sullivan M, Snippes P, Kuskowski MA, Smith KE (2003). Isolation and molecular characterization of nalidixic acid-resistant extraintestinal pathogenic *Escherichia coli* from retail chicken products. Antimicrob Agents Chemother.

[18] Spurbeck RR, Dinh PC, Walk ST, Stapleton AE, Hooton TM, Nolan LK, Kim KS, Johnson JR, Mobley HLT (2012). *Escherichia coli* isolates that carry *vat*, *fyuA*, *chuA*, and *yfcV* efficiently colonize the urinary tract. Infect Immun.

[19] Stumpe S, Schmid R, Stephens DL, Georgiou G, Bakker EP (1998). Identification of OmpT as the protease that hydrolyzes the antimicrobial peptide protamine before it enters growing cells of Escherichia coli. J Bacteriol.

[20] Johnson TJ, Wannemuehler YM, Nolan LK (2008). Evolution of the iss gene in Escherichia coli. Appl Environ Microbiol.

[21] Skjøt-Rasmussen L, Olsen SS, Jakobsen L, Ejrnæs K, Scheutz F, Lundgren B, Frimodt-Møller N, Hammerum AM (2013). Escherichia coli clonal group a causing bacteraemia of urinary tract origin. Clin Microbiol Infect.

